# Well-being Assessment of Medical Professionals in Progressive Levels of Training: Derived from the WHO-5 Well-being Index

**DOI:** 10.7759/cureus.3790

**Published:** 2018-12-28

**Authors:** Wasique Mirza, Annina M Mirza, Muhammad Sabih Saleem, Pravin P Chacko, Maryyam Ali, Muhammad Nauman Tarar, Afia Babar, Jeremy Freiwald, Konstantin Talitskiy

**Affiliations:** 1 Internal Medicine, Geisinger Commonwealth School of Medicine, Scranton, USA; 2 Psychology, The University of Scranton, Scranton, USA; 3 Internal Medicine, Shifa International Hospital, Islamabad, PAK; 4 Internal Medicine, The Wright Center for Graduate Medical Education, Scranton, USA

**Keywords:** resident training, quality improvement, physician burnout, well-being index, physician well-being, student loans, financial stress, college expense, who-5 well-being index, who-5

## Abstract

The provision of quality health care is of utmost importance for a physician. Over the years, there has been much debate regarding work-life imbalance and physician burnout, which may, in turn, have adverse effects on the quality of care. Medical school students, residents, interview candidates for residency, and internal medicine faculty are all under a varying degree of stress, which may impact their personal and professional lives. We distributed questionnaires to investigate our hypothesis: Progression in training years leads to a decline in well-being. The main objective of our assessment was to help devise interventions to improve the quality of training and the productivity of internal medicine physicians. Understanding the emotional functioning of physicians will help us improve the learning environment and, in turn, have a positive impact in the future for medical professionals. Medical students are burdened with excessive loans for undergraduate and graduate studies, which contributes to higher rates of burnout, depression, and suicide among medical professionals, which can lead to a direct and negative impact on quality of care. Our study showed that well-being scores declined with increasing financial stress; they were also affected by the visa status and training background of our subjects as medical students.

## Introduction

The World Health Organization-Five (WHO-5) Well-Being Index is a short, self-administered questionnaire covering five positively worded items, related to positive mood (good spirits, relaxation), vitality (being active and waking up fresh and rested), and general interests (being interested in things). It has shown to be a reliable measure of emotional functioning and a good screening tool for depression. Administering the WHO-5 Well-Being Index takes a few minutes [1-2]. Although WHO-5 was developed without any diagnostic specificity [3], the clinical validity of WHO-5 was assessed to be high, as the scale can be used regardless of underlying illness and across many different settings [4]. Adaptations from the score have been utilized to assess patient wellness as well as employee wellness in research. With physician burnout being increasingly identified as a growing concern, we decided to use this scale to measure the stress and general well-being of physicians during progressive levels of training. There has been a growing concern for work-life imbalance and physician burnout leading to adverse effects on the quality of patient care. During the years of residency training, the Liaison Committee on Medical Education (LCME) and Accreditation Council for Graduate Medical Education (ACGME) have specified work hours and patient-care caps to keep the workload in check, but there are many other factors that affect the well-being of medical students and residents. In addition, after their training is complete, such caps and safety measures simply do not exist in medical practice, depending on the practitioners themselves to police their own work-life balance. Medical students are dealing with study-related stress, and a constant barrage of new information and the uncertainty of future decisions play a role. For new interns, a new system, environment, and adjustment to the routine and responsibility can affect vitality and well-being. On the other hand, a senior resident may be affected by upcoming job uncertainty and increasing responsibility. Faculty members, while on a career path, are dealing with increasing responsibilities and the stress surrounding a growing family. While within a residency training program, semiannual mentor meetings address these issues as well. We recognize a need to assess this issue and scale the well-being with the goal of designing an intervention to improve the training experience for residents. Our main objectives were two-fold. The first was to study the medical student's and physician's well-being with each progressive year and understand its distribution between sexes. This assessment will help guide any interventions, if needed, for improving the quality of training and productivity of internal medicine physicians. Second, during the years of training, progressing from intern to senior residents, the distribution of stress is influenced by workload, block rotations, and background training experiences and compounded by factors such as marital status, children, visa status, and financial stress from student loans. This would help us understand the emotional functioning at progressing levels of training and devise future strategies based on the results to improve the learning environment and, in turn, positively affect the future of medicine.

## Materials and methods

**Figure 1:**
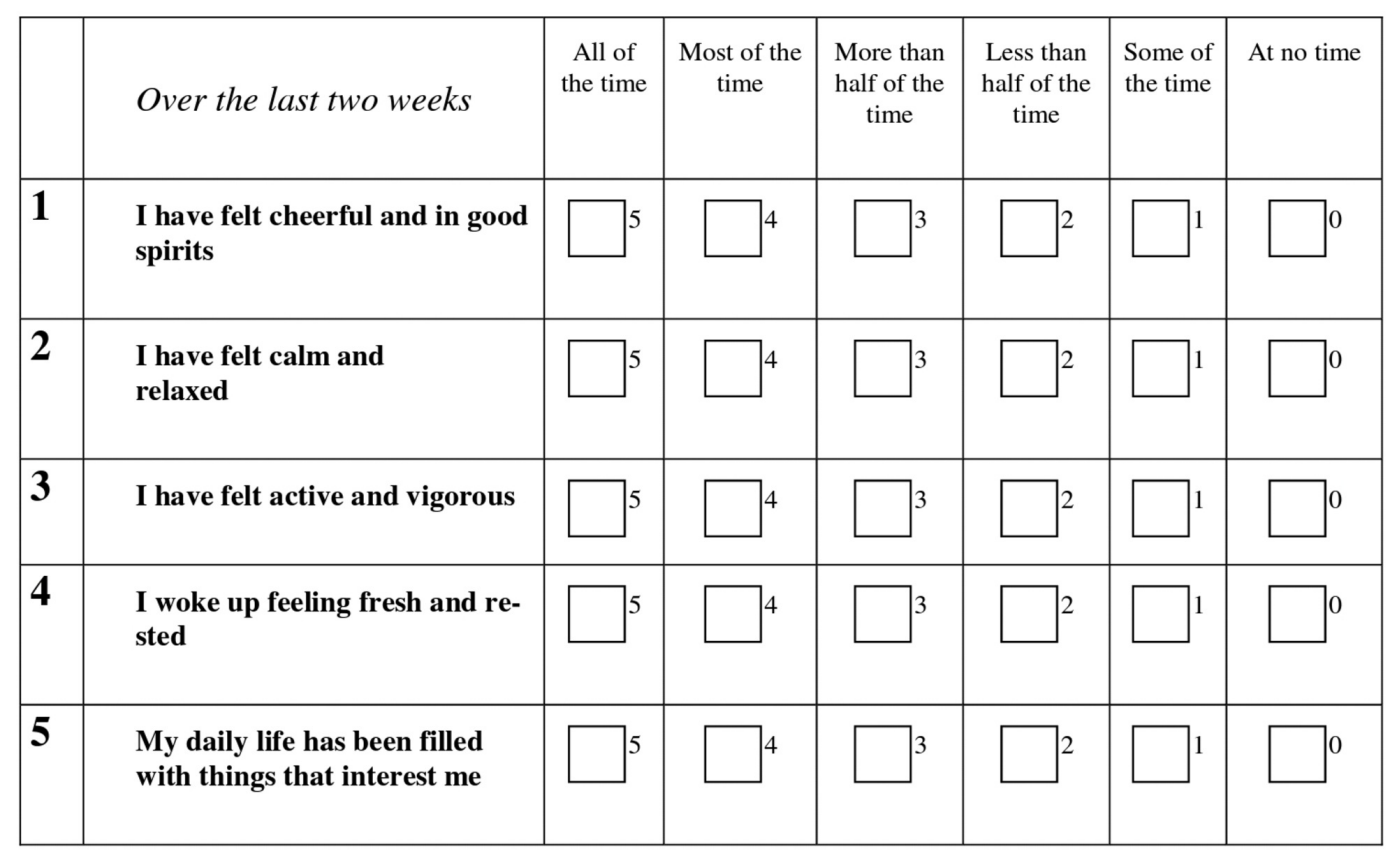
WHO-5 well-being index This figure has been taken from reference 1, with minor modifications. (WHO-5: World Health Organization-Five)

A cross-sectional study was carried out, where we distributed questionnaires (sample is shown in Figure 1) to medical students (MS-3 and MS-4) at Geisinger Commonwealth School of Medicine; transitional physicians recruited from internal medicine residency candidates (all three years); interview candidates for the match year 2017; and internal medicine faculty. The questionnaire was distributed through Survey Monkey and incomplete responses were excluded.

WHO-5 is a highly practical tool that can be applied in both clinical practice, as well as in research studies, to assess well-being over time or to compare well-being between groups [1]. Identifying data, such as Social Security Number (SSN), name, or date of birth, wasn’t utilized. The purpose of the study was non-commercial and the data will only be used for scientific publication. The data was collected by anonymous surveys and no identifier, except for general questions, such as the year of training, sex, and marital status, was used. The data were only saved on one computer with the primary investigator. We formulated a hypothesis. Progression in training years leads to a decline in well-being. This hypothesis suggested that medical students will show the highest scores on the questionnaire, indicating the highest well-being among the groups. This will decline with the progressive level of training, leading to the lowest scores among practicing faculty members.

The raw score was calculated by summating the scores from the five answers and had a range between 0 and 25 (0: worst possible; 25: best possible quality of life). To obtain a percentage score ranging from 0 to 100, the raw score was multiplied by four. A percentage score of 0 represents the worst possible, whereas a score of 100 represents the best possible quality of life. Evidence suggested that a score of 50 (cut-off score) or below was indicative of low mood, though not necessarily depression. In order to monitor possible changes in well-being after interventions, a 10% difference could be regarded as a significant change. From the average scores of each group based on the criteria above, we compared data using a simple univariate raw analysis. A single/one sample t-test was used to get an indication of the total sample well-being. A comparison between groups was done by an analysis of variance (ANOVA).

## Results

**Figure 2:**
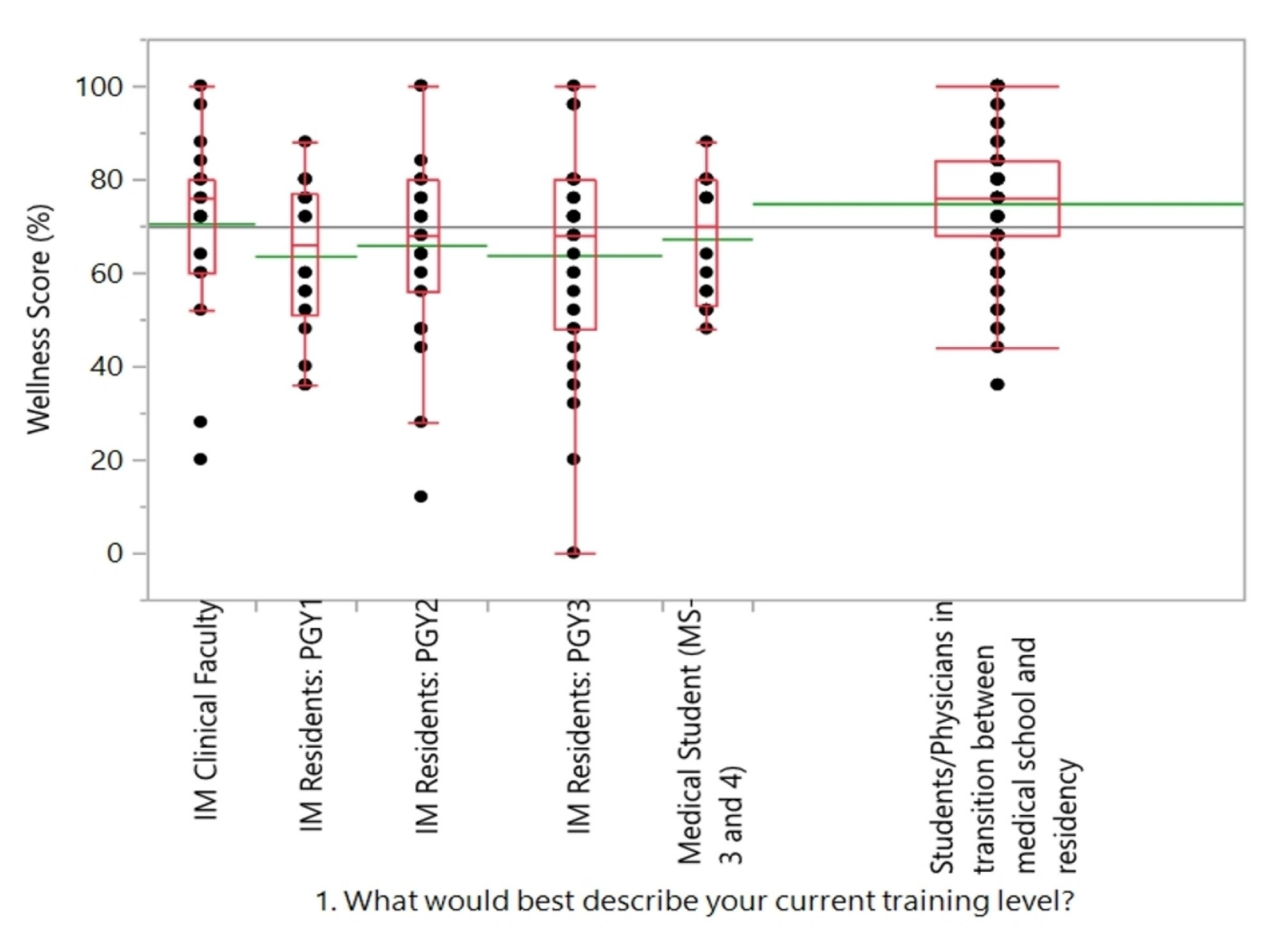
Level of training vs wellness score

The total number of people who responded were 195. Of the 195 respondents, 88 (45%) were medical students/physicians in transition between medical school and residency, 71 (36%) were internal medicine residents (9%: post-graduate year 1 (PGY1), 12% PGY2, and 15% PGY3, respectively), 16 (8%) were medical students (third and fourth years), and we had 20 (10%) respondents who belonged to the internal medicine clinical faculty. The mean wellness score (percentage) for all respondents was 69.8%. Physicians/ medical students in transition between medical school and residency had the highest mean score of 74.8%, followed by clinical faculty (70.5%), and that of internal medicine residents was 64.3% (with PGY1 residents averaging the lowest score of 63.5%, PGY2 averaging 65.9%, and PGY3 being 63.7%; Figure 2).

**Figure 3:**
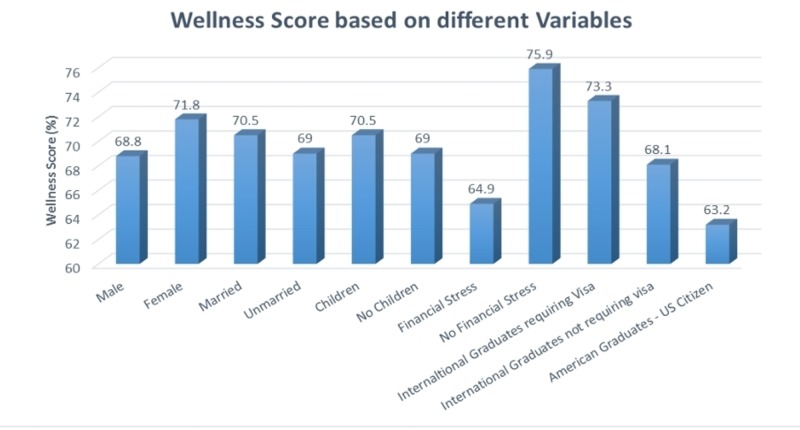
Wellness score based on different variables

Of the total respondents, we had 125 (64%) males vs 70 (36%) females. Of the total population, 113 (58%) were married, 57 (29%) had children. 94 (48%) of respondents were international graduates requiring a visa, 72 (37%) were international graduates not requiring a visa, and 29 (15%) were American graduates not requiring a visa. Our data showed that a total of 107 (55%) people responded “yes” to financial stress. The mean wellness score for females (71.8%) was slightly higher than for males (68.8%). Married people had a marginally higher mean score (70.5%) as compared to singles (69%). Physicians and students with children had a slightly higher mean score (71%) vs no children (69.4%). Students and physicians with no financial stress had a significantly higher mean score (75.9%) as compared to respondents who reported financial stress (64.9%). International graduates requiring a visa had the highest mean score (73.3%) followed by US citizen international graduates (68.1%) and American graduates with US citizenship scoring the lowest (63.2%) (Figure 3).

**Figure 4:**
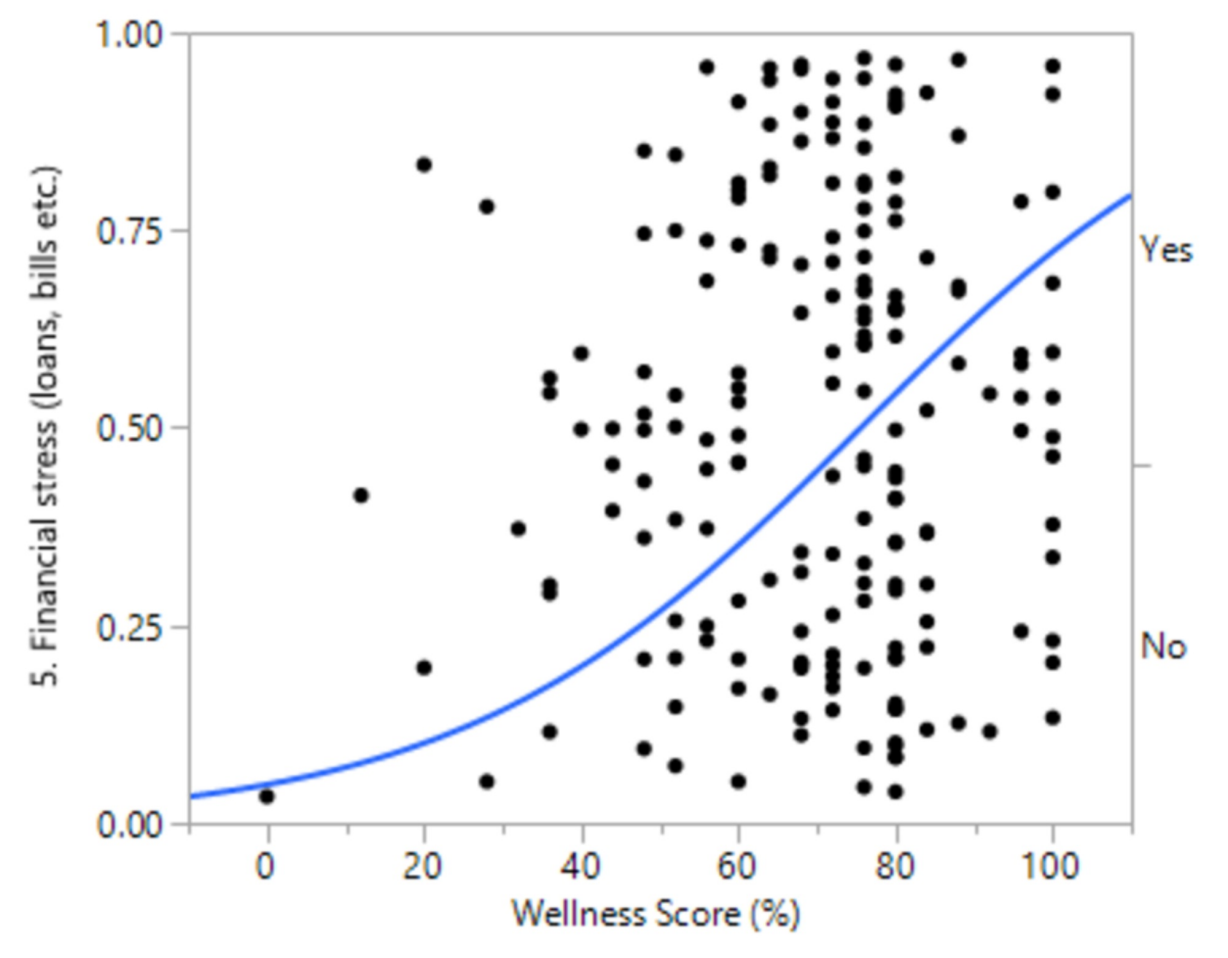
Financial stress and wellness score

**Figure 5:**
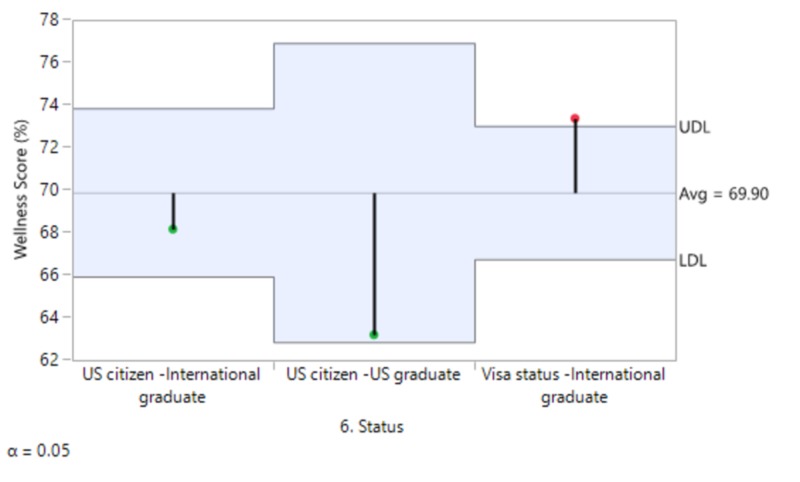
Visa status and wellness score

Although females tended to have a higher wellness score than males, as did married people and people with children, the difference was not significant. There were also no significant differences between different PGY levels. We did, however, find a significant difference in the wellness score based on financial stress, as the difference was >10%. People who answered “Yes” to financial stress were more likely to have a lower wellness score (odds ratio (OR) 1.04, CI 1.02-1.06, p<0.001). There was also a significant difference between international graduates on a visa and US citizen American graduates (10%). International graduates on a visa were more likely to have a higher wellness score than their US citizen American graduate counterparts (Figures 4-5].

## Discussion

In the life cycle of a physician, while entering medical school is the accomplishment of a dream, it is only the beginning of a long journey, culminating in the completion of residency training and then embarking on a lifelong quest for knowledge and professional excellence. One realizes very quickly that this is not an easy task. The stresses that add to the struggles of achieving these goals multiply with every step in the right direction. The joy of triumph is often complicated by the addition of several factors covering a broad spectrum from economic, social, as well as personal, leading to a profound impact on well-being. Studying for such a demanding task in itself is stressful enough; however, with age and accomplishments come maturity, wisdom, as well as responsibilities. The least of which is the burden that comes with making decisions that may affect one's life for decades to come. Budding physicians are no longer in the shadows or protected by the decisions of their parents. They are in charge of their life decisions, including the burden of financial responsibility. Educational loans have become the single most stressful factor that affects the well-being of college students and continues even after they graduate and reach financial independence. Working in the new and demanding environments of today's healthcare, dealing with colleagues, case managers, and hospital officials, with varying backgrounds, styles, and egos, is not an easy task by any means. While learning to master the electronic health record (EHR) alone is capable of leading to sleepless nights, it is a challenge getting acclimatized to the exhaustive reporting requirements of the parent institutions, and the whimsical data-mining metrics can overwhelm one’s day; at times, relegating patient care to a distant third, leading to those moments that make one wonder about the purpose of all this. When we embarked on this study, our thought process was that the stress level changes as physicians pass through different phases of development and responsibility. We wanted to see if there was a linear progression or regression in stress and, conversely, in well-being over the years and to identify any specific factors that have an additive effect on the overall state of well-being. We chose to use the WHO-5 Well-Being Index as a tool to study this population due to various reasons: ● It is free and available in the public domain. ● It is short and easier to complete. ● It provides valuable insight without adding an undue additional burden of paperwork on study participants. The original WHO-5 Index focuses on the past two weeks. We made the modification to expand the inquiry to over six months in order to avoid any biases associated with transient and rotation-related stresses. According to a systematic review, the WHO-5 has been used to analyze a wide variety of aspects [1], including coping strategies [5], well-being in occupational health settings [6], the association between workplace stress and well-being [7], the links between working condition and well-being [8], as well as the association between psycho-social conditions and well-being [9]. We hypothesized that the stress level and the resulting negative impact on well-being will be the lowest in the early formative years, i.e., medical school and early residency, and will increase as one is further along in their training and career. The results of the study did not show any statistically significant difference in the overall levels of well-being over the years. However, it did show minor differences, such as the lowest levels in well-being scores in medical students and PGY2 residents. Studying the well-being scores based on demographic variables, again, there were no statistically significant differences except in two categories. Groups were divided based on: ● Sex ● Marital Status ● Children/No Children ● Financial Stress ● US/Foreign Medical Graduates ● Visa Status Although females tended to have a higher wellness score than males, as did married people and people with children, the difference was not significant. There was also no significant differences between different PGY levels. We did, however, find a significant difference in the well-being score based on financial stress, as the difference was >10%. People who answered “Yes” to financial stress were more likely to have a lower well-being score (OR 1.04, CI 1.02-1.06, p<0.001). There was also a significant difference between international graduates on a visa and US citizen American graduates (10%). International graduates on a visa were more likely to have a higher well-being score than their US citizen American graduate counterparts. Both of the statistically significant findings can be directly attributed to financial stress. While there was a clear difference in the well-being scores of those who had financial stress (64.9) as compared to those who did not (75.9), it can be extrapolated from the comparison between US and foreign graduates that financial stress is heavily influenced by the escalation and accumulation of student loans. International graduates, in general, do not have any or, at most, minimal student loans. Once they are in a residency program, they are well on their way to financial security and independence. In contrast, US graduates are burdened with hundreds of thousands of dollars in student loans to pay over the first several years of their professional careers. Foreign medical graduates with visa concerns fared better than US grads, however, that statistic is affected by a mix of international graduates from different international schools. While US citizen and green-card-holding graduates from most international medical schools do not have significant student loans, there is a growing number of graduates from Caribbean-based medical schools. Their numbers have increased over the past decade and a half to become the second-largest group of foreign medical graduates after India. Most of these graduates are US citizens and while they do not have visa-related issues, they are still burdened with student loans of undergraduate and graduate studies comparable to US graduates.

Our goal with the information obtained from this small study was to identify and study significant points in time, leading to the development of strategies to slow this progression. With focused interventions, we can plan small changes to improve physician well-being, which may be the starting point for a reduced incidence of future burnout. With residency training stress acknowledgment and planned interventions, we can help improve the overall learning experience.

## Conclusions

While one would think that the nature of stresses through medical training and beyond will result in a linear progression or regression of well-being, our study did not show that. However, the results did show a relationship between financial stress and low well-being scores. Since our study was limited to physicians and physicians in training, we do not know how the baseline scores obtained compare with those in similar phases of other professions. Wellness data from various other studies have shown lower wellness scores, higher rates of burnout, and an alarmingly higher rate of depression and suicide among medical professions. More in-depth studies will be needed to further investigate those differences in order to identify profession-specific concerns. A concerted effort is needed to target such stress spots to improve the overall well-being of our medical professionals and harness the alarmingly increasing rates of burnout seen in the medical community.
